# Effect of Antisolvent Used to Regenerate Cellulose Treated with Ionic Liquid on Its Properties

**DOI:** 10.3390/molecules29174227

**Published:** 2024-09-06

**Authors:** Marta Bloch, Magdalena Woźniak, Krzysztof Dwiecki, Sławomir Borysiak, Izabela Ratajczak

**Affiliations:** 1 Department of Chemistry, Faculty of Forestry and Wood Technology, Poznan University of Life Sciences, Wojska Polskiego 75, 60625 Poznan, Poland; marta.babicka@up.poznan.pl (M.B.); izabela.ratajczak@up.poznan.pl (I.R.); 2 Department of Food Biochemistry and Analysis, Faculty of Food Science and Nutrition, Poznan University of Life Sciences, Mazowiecka 48, 60623 Poznan, Poland; krzysztof.dwiecki@up.poznan.pl; 3 Institute of Chemical Technology and Engineering, Poznan University of Technology, Berdychowo 4, 60965 Poznan, Poland; slawomir.borysiak@put.poznan.pl

**Keywords:** solvolysis, 1-ethyl-3-methylimidazolium acetate, crystallinity, nanocellulose

## Abstract

The solvolysis reaction with ionic liquids is one of the most frequently used methods for producing nanometer-sized cellulose. In this study, the nanocellulose was obtained by reacting microcrystalline cellulose with 1-ethyl-3-methylimidazolium acetate (EmimOAc). The aim of this research was to determine the influence of various antisolvents used in the regeneration of cellulose after treatment with ionic liquid on its properties. The following antisolvents were used in this research: acetone, acetonitrile, water, ethanol and a mixture of acetone and water in a 1:1 *v*/*v* ratio. The nanocellulose was characterized by Fourier transform infrared spectroscopy (FTIR), X-ray diffraction (XRD), dynamic light scattering (DLS), scanning electron microscopy (SEM) and elemental analysis (EA). The results show that the antisolvent used to regenerate cellulose after the solvolysis reaction with EmimOAc affects its properties. Water, ethanol and a mixture of acetone and water successfully removed the used ionic liquid from the cellulose structure, while acetone and acetonitrile were unable to completely remove EmimOAc from the cellulosic material. The results of the XRD analysis indicate that there is a correlation between the ionic liquid content in the regenerated cellulose and its degree of crystallinity. Among the tested solvents, water leads to the effective removal of EmimOAc from the cellulose structure, which is additionally characterized by the smallest particle size and non-formation of agglomerates.

## 1. Introduction

Nanocellulose is of great interest in the design of modern materials that are expected to be both efficient and environmentally friendly [1]. As a natural biopolymer obtained from biomass, including waste biomass, nanocellulose fits perfectly into this trend. Renewable sources for the production of nanocellulose may include, among others, cotton, bamboo, the straw of various plant species and wood [2,3].

There are three basic types of nanocellulose: cellulose nanocrystals (CNCs), cellulose nanofibers (CNFs) and bacterial nanocellulose (BNC) [4]. Despite their similar chemical composition, these forms differ in their physical properties, such as morphology, crystallinity and particle size [5]. The properties of nanocellulose are significantly influenced by the production method used as well as the source of lignocellulosic material [1]. Nanocellulose is characterized by many favorable properties, including high surface area, mechanical strength, optical transparency, low density, biocompatibility and surface functionalization potential [5,6]. Thanks to this unique combination of properties, it is used in various fields, such as healthcare [7], food packaging [6], polymer composites [8,9], electronics [10] and construction [11].

A wide spectrum of nanocellulose production techniques is currently known, ranging from conventional methods (mechanical or chemical methods) to more environmentally friendly approaches. The more innovative and bio-friendly methods of producing nanometric cellulose include, among others, enzymatic hydrolysis and reactions with deep eutectic solvents or ionic liquids [1]. The use of ionic liquids (ILs) for the production of nanocellulose is of great interest, as evidenced by the number of publications on this topic, which has exceeded a thousand in the last decade [12]. Ionic liquids are compounds with an ionic structure and a melting point below 100 °C, which usually consist of a large organic cation and a smaller anion, which may be organic or inorganic. These compounds are characterized by many beneficial properties such as thermal and chemical stability, non-volatility, non-flammability, low vapor pressure and the ability to dissolve lignocellulosic biomass [13,14]. Ionic liquids, due to their ability to dissolve lignocellulosic biomass and the potential for recycling and reuse, have become a more economical and safer alternative for the production of natural nanomaterials compared to conventional methods, such as acid hydrolysis, where concentrated acids are used [12]. The literature reports indicate that the properties of nanocellulose are influenced by both the cation and anion of the ionic liquid used in the process [15,16,17]. Various ionic liquids are applied to obtain nanocellulose, but the most frequently used include 1-butyl-3methylimidazolium hydrogen sulfate [Bmim][HSO_4_], 1-ethyl-3-methylimidazolium acetate [Emim][OAc] and 1-ethyl-3-methylimidazolium chloride [Emim][Cl] [18,19]. The antisolvent used to regenerate cellulose from ionic liquids also plays an important role in the process of obtaining nanometer-sized cellulose and influences its properties [20,21]. The antisolvents used in nanocellulose production include water, alcohols (methanol, ethanol, propanol), acetonitrile and acetone [20,21]. The results from a molecular simulation study performed by Gupta et al. [22] indicated that water was a more effective antisolvent than ethanol and acetone in the regenerative effect of cellulose from cellulose/1-*n*-butyl-3-methylimidazolium acetate mixture.

Taking into account the fact that the properties of nanocellulose are affected by both the type of ionic liquid used and the antisolvent applied for cellulose regeneration, research aimed at determining the effect of both the ionic liquid and antisolvent on the properties of the obtained nanomaterial seems justified. Therefore, the aim of this study was to determine the effect of various antisolvents (acetone, acetonitrile, ethanol, water and acetone/water mixture) used in the regeneration of cellulose after treatment with ionic liquid, namely 1-ethyl-3-methylimidazolium acetate, on its properties.

## 2. Results and Discussion

### 2.1. Infrared Spectroscopy (ATR-FTIR)

**Figure 1:**
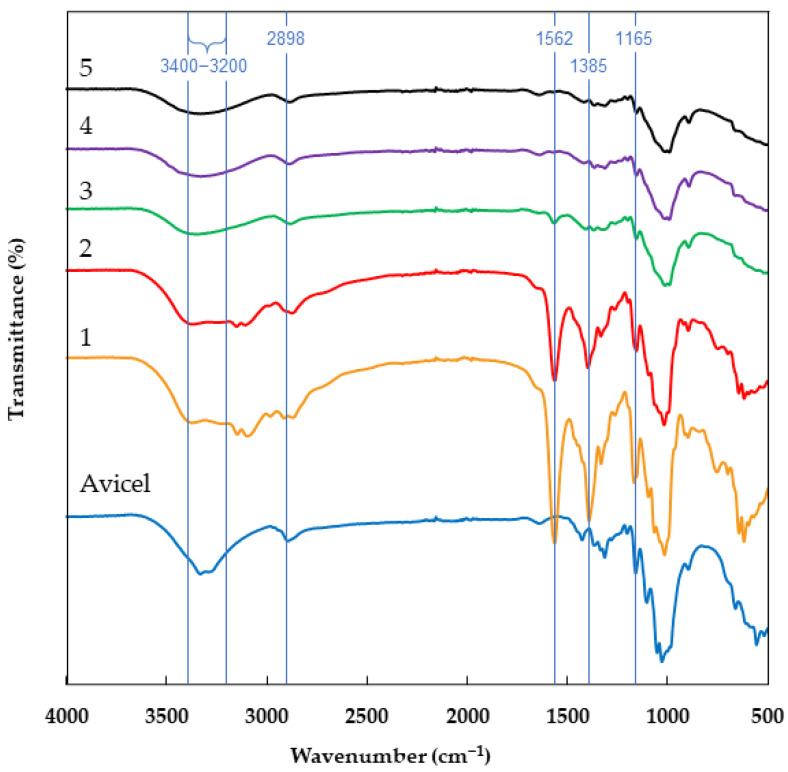
FTIR spectra of Avicel cellulose (Avicel) and cellulose regenerated with various antisolvents: 1—acetone, 2—acetonitrile, 3—ethanol, 4—water, 5—acetone/water (1:1 *v*/*v*).

The structure of the cellulose materials obtained after solvolysis reaction with ionic liquid and regenerated using various antisolvent variants was analyzed by Fourier transform infrared spectroscopy and the spectra of the tested samples are presented in Figure 1.

The spectra of all cellulose samples before and after treatment with ionic liquid contained bands characteristic for cellulosic material, including a broad band in the region 3400–3200 cm^−1^ assigned to the stretching vibrations of the hydrogen bond O-H [23,24]. In the spectra of the cellulose samples, especially in the case of samples 3, 4, and 5, the intensity for this band decreased compared to the band intensity of the Avicel sample, which may be related to the reduction in the number and strength of O-H bonds in these samples [25,26]. The spectra of all cellulose materials contained bands at 2898 cm^−1^ attributed to the C-H stretching vibrations [27], 1370 cm^−1^ related to C-H bending vibration [8,28], 1165 cm^−1^ originated from the C-O anti-symmetric bridge stretching, 1030 cm^−1^ related to the C-O-C bending vibrations [29] and 895 cm^−1^ associated with the glycoside C-H deformation with ring vibration and -OH bending of β-glycoside linkages between glucose in cellulose [23,26]. Moreover, in the spectra of cellulose regenerated with acetone (1) and acetonitrile (2), the bands were visible at 1562 cm^−1^ (C=N stretching vibration), 1385 cm^−1^ (C-H bending vibration) and 1165 cm^−1^ (C=O stretching vibration), originating from EmimOAc [30], which suggests that the ionic liquid was not completely removed from the cellulose structure. In the spectra of cellulose samples 1 and 2, additional peaks also appeared in the range of 3400–3000 cm^−1^, which can be related to the overlapping bands of the stretching vibrations of hydrogen groups and C-H vibrations of the imidazolium ring of EmimOAc [31,32]. The spectrum of cellulose regenerated with ethanol (3) showed a band with low intensity at 1562 cm^−1^ (C=N stretching vibrations), which suggests that a small amount of ionic liquid was not removed from the cellulose structure. In the case of the spectra of cellulose samples regenerated using other antisolvents (water and acetone/water), no bands indicating the presence of 1-ethyl-3-methylimidazolium acetate were observed. Mahadeva and Kim [20] found that cellulose treated with EmimOAc and regenerated with methanol, water and isopropanol/water mixture contained a peak in the FTIR spectrum at 1562 cm^−1^, attributed to the C=N stretching vibrations of the ionic liquid, which were associated with the remaining ionic liquid in the structure of the obtained material. In contrast, the FTIR spectra of methanol/water-washed cellulose did not contain this peak, suggesting that the IL was successfully removed from the cellulose with this antisolvent [20]. Elhi et al. [21] found that, of the three antisolvents (water, acetone, ethanol) used to regenerate IL-treated cellulose, acetone retained large amounts of IL in cellulose, especially when EmimOAc was used in the hydrolysis reaction. Also, a certain amount of ionic liquid was observed in ethanol-regenerated cellulose compared to the water-regenerated cellulose sample, which had no traces of IL [21].

### 2.2. Elemental Analysis (EA)

The nitrogen concentration in the cellulose samples determined by elemental analysis is given in Table 1.

The results of the nitrogen concentration in the tested samples confirm the effective removal of the ionic liquid used from the cellulose structure, where ethanol (3), water (4) and acetone/water mixture (5) were used as antisolvents. However, in the case of cellulose regenerated with ethanol (3), the nitrogen concentration was slightly higher (although statistically insignificant) than in the cellulose samples 4 and 5, which is consistent with the FTIR results. In turn, the cellulose samples regenerated with acetone and acetonitrile were characterized by high nitrogen content, indicating that EmimOAc was not removed from these materials. The obtained results show that the effectiveness of removing the used ionic liquid from the cellulose structure depends (using the same volume) on the antisolvent used for its regeneration. The results presented by Mahadeva and Kim [20] also indicated that the degree of ionic liquid removal from the IL-treated cellulose structure depends on the antisolvent used. The nitrogen content obtained by these authors in cellulose samples regenerated with various antisolvents ranged from 0.75% (methanol/water) to 2.67% (water). However, the nitrogen content results obtained by these authors are opposite to those presented in this study, because in these studies water turned out to be the most effective antisolvent for removing IL from the cellulose structure, which may be caused by different conditions of cellulose treatment.

### 2.3. X-ray Diffraction (XRD)

**Figure 2:**
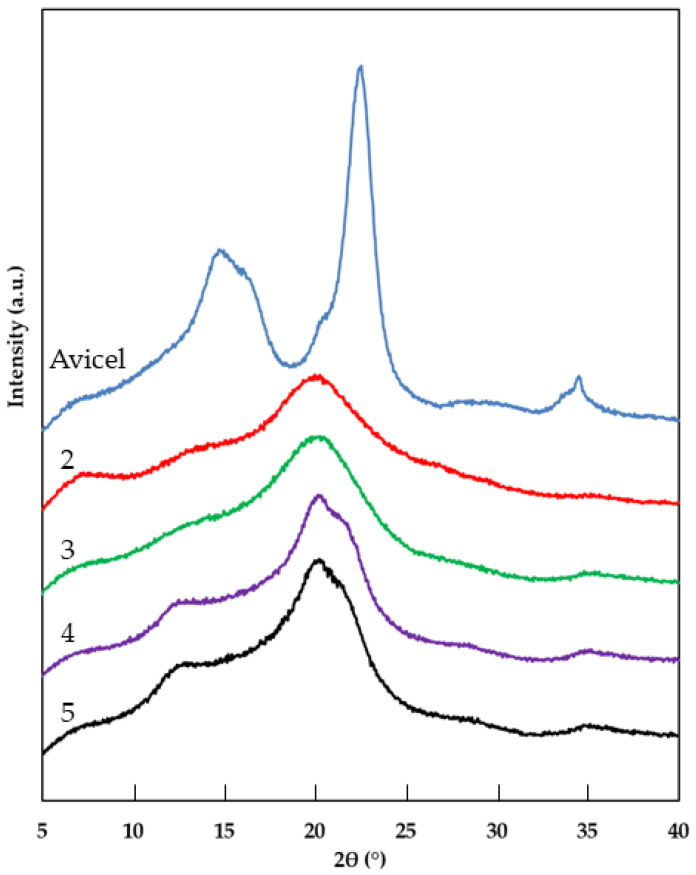
XRD patterns of Avicel cellulose and EmimOAc-treated cellulose regenerated with different antisolvents: 2—acetonitrile, 3—ethanol, 4—water, 5—acetone/water (1:1 *v*/*v*).

The diffraction patters of Avicel cellulose and cellulose materials treated with EmimOAc and washed with various antisolvents are presented in Figure 2.

The acetone-regenerated nanocellulose sample was characterized by high viscosity, which made XRD, DLS and SEM analyses impossible. The high viscosity of the sample was related to the fact that the ionic liquid was not removed from the cellulose structure, which was confirmed by FTIR (Figure 1) and elemental analysis (Table 1).

The XRD diffraction profile of Avicel cellulose contained three well-defined peaks at 2θ = 15° assigned to $\bar{11}0$ plane, 2θ = 17° ascribed to 110 and 2θ = 22.6° corresponding to the 200 plane [33,34]. The presence of these peaks in the Avicel XRD pattern indicates the crystalline structure of cellulose I. In contrast, the diffraction patterns of nanocellulose samples 3 and 4 contained two peaks at 2θ = 20.0° and 2θ = 21.6°, which correspond to the 110 and 200 planes, respectively [15,35,36]. The presence of these peaks indicates the formation of cellulose II. The diffraction patterns of nanocellulose samples 2 and 3 exhibited a peak at 2θ = 21.6°, corresponding to the overlapping 100 and $\bar{11}0$ planes, which is characteristic of cellulose III [33]. The obtained X-ray diffraction patterns demonstrate that the use of 1-ethyl-3-methylimidazolium acetate resulted in a polymorphic transformation of cellulose I, indicating that the antisolvent employed for the regeneration of nanocellulose influences the polymorphic structure of the cellulose material. Polymorphic transformation of cellulose under the influence of ionic liquids, including EmimOAc, is a well-documented phenomenon in the literature and depends on various factors, including the reaction conditions and the type of cellulose material used [15,37,38].

The solvolysis reaction of cellulose with EmimOAc also affects the ratio of the crystalline and amorphous portions of cellulose, resulting in differences in the crystallinity index (Xc), as shown in Table 2.

In all analyzed cellulose samples, a significant decrease in the crystallinity index was observed compared to Avicel cellulose, and these values varied depending on the antisolvent used. The crystallinity index of cellulose regenerated using various antisolvents decreased in the following order: acetone/water > water > ethanol > acetonitrile, which indicated that the polarity of the antisolvent played an important role in reducing the number of crystallinity areas in the material. The use of a lower-polarity antisolvent for the regeneration of cellulose resulted in a decrease in the Xc value. The highest reduction in Xc value of EmimOAc-treated cellulose compared to Avicel cellulose was observed for cellulose sample 2 (acetonitrile), which was 56%, while the lowest decrease in crystallinity index of 38% was found for cellulose sample 5 (acetone/water). The decrease in the crystallinity index observed in cellulose samples treated with ionic liquid is characteristic of cellulosic materials in which the process of transformation of cellulose I into cellulose II was found [36]. Literature data have previously reported a decrease in the degree of crystallinity of cellulose after the solvolysis reaction with various ionic liquids [25,38,39]. Moreover, literature reports confirm that the antisolvent used in the regeneration of nanocellulose affects the crystallinity index of the obtained cellulosic material [20]. The crystallinity of cellulose treated with 1-butyl-3-methylimidazolium chloride and regenerated using different antisolvents increased in the following order: water < methanol < ethanol < n-propanol [40]. The results of the XRD analysis indicate that there is a correlation between the ionic liquid content (Table 1) in the regenerated cellulose and its degree of crystallinity (Table 2), which is consistent with the literature data [20].

### 2.4. Dynamic Light Scattering (DLS)

**Figure 3:**
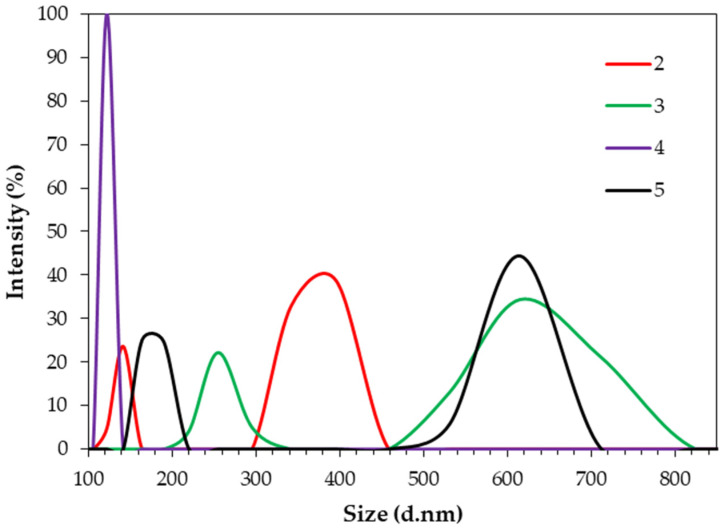
The average particle size of the cellulose treated with EmimOAc and regenerated with different antisolvents: 2—acetonitrile, 3—ethanol, 4—water, 5—acetone/water (1:1 *v*/*v*).

The average particle size (hydrodynamic diameter) of the cellulose samples after the solvolysis reaction with EmimOAc, assessed by dynamic light scattering (DLS), is shown in Figure 3.

The results of DLS analysis indicated that all EmimOAc-treated cellulose samples were characterized by a lower average particle size than that of Avicel cellulose, which was in the range of 1300–4800 nm. The cellulose regenerated with water contained the smallest average particle size, which did not exceed 200 nm. This sample was characterized by the highest sample uniformity and no agglomerate formation, as indicated by one narrow, high-intensity peak. The remaining cellulose samples showed the presence of two particle size fractions with different values of average particle diameter. The acetonitrile-washed cellulose contained one fraction with an average particle size below 200 nm and a second fraction with a particle size ranging from 300 to 450 nm. Cellulose regenerated using other antisolvents (ethanol and mixture of acetone and water) was characterized by a larger particle size and the presence of two fractions differing in particle size. The result of the average particle diameter suggests that cellulose washed with the antisolvents used, except water, formed agglomerates. The formation of agglomerates by cellulose after ionic liquid treatment has been previously reported in the literature [41,42]. Liu et al. [43] indicated that the morphology of cellulose treated with 1-butyl-3-methylimidazolium acetate and regenerated using conventional antisolvent, including acetonitrile, water and acetone, resembles massive and agglomerate texture. A molecular simulation study presented by Gupta et al. [22] demonstrated that, among the three tested antisolvents (water, acetone, ethanol), water is the most effective at breaking the hydrogen bonds between cellulose and acetate anions from the BmimOAc.

### 2.5. Scanning Electron Microscopy (SEM)

**Figure 4:**
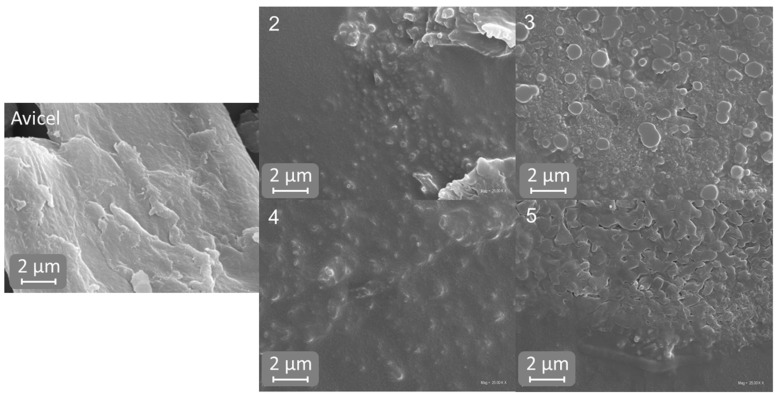
The SEM images of the Avicel cellulose and cellulose treated with EmimOAc and regenerated with different antisolvents: 2—acetonitrile, 3—ethanol, 4—water, 5—acetone/water (1:1 *v*/*v*).

The morphology of cellulosic materials after EmimOAc solvolysis and regeneration using various antisolvents was examined by SEM analysis, and the results in the form of images are presented in Figure 4.

The treatment of microcrystalline cellulose with EmimOAc resulted in a reduction in the particle size of the obtained cellulose, which is confirmed by the SEM images shown in Figure 4. Moreover, the SEM images show that the shape of the regenerated cellulose depended on the type of antisolvent used for this process. The cellulose washed with acetonitrile (2) and ethanol (3) showed a more regular and spherical structure than other cellulose samples. Moreover, all cellulose materials, regardless of the antisolvent used for its regeneration, showed the formation of agglomerates, which is consistent with the DLS analysis (Figure 3). Mahadeva and Kim [20] found that cellulose treated with EmimOAc and washed with different antisolvents (methanol, water, methanol/water mixture and isopropanol/water mixture) exhibited an aggregated and layered structure, with the layer thickness depending on the type of antisolvent.

## 3. Materials and Methods

### 3.1. Materials

Microcrystalline cellulose Avicel PH-101 with ~50 µm particle size and ionic liquid—1-ethyl-3-methylimidazolium acetate (EmimOAc) were purchased from Sigma Aldrich (Darmstadt, Germany). All solvents used in the research were of analytical grade and purchased from Sigma Aldrich (Darmstadt, Germany). Merck Millipore grade deionized water purified in the Milli-Q system (Millipore, Bedford, MA, USA) was used.

### 3.2. Nanocellulose Preparation

Microcrystalline cellulose was mixed with EmimOAc at a 1:5 weight ratio under intense stirring using a heating mantle with magnetic stirring (ChemLand, Stargard, Poland) for 10 min at 110 °C, without the addition of solvents based on the method described in our previous research [15]. The solvolysis reaction was performed six times, and six different antisolvent variants were used to regenerate the nanocellulose: (1) acetone, (2) acetonitrile, (3) ethanol, (4) water and (5) a mixture of acetone and water in a volume ratio of 1:1, which resulted in five different nanocellulose samples numbered according to the antisolvents. A total of 300 mL of each antisolvent was used to regenerate nanocellulose. The final cellulose products were filtered and dried at room temperature and next over P_2_O_5_ (Merck KGaA, Darmstadt, Germany).

### 3.3. Characterization of Cellulose

#### 3.3.1. Infrared Spectroscopy

The cellulose samples were analyzed using the attenuated total reflectance (ATR) Fourier transform infrared spectroscopy (FTIR). The spectra of the samples were recorded by a Nicolet iS5 spectrophotometer (Thermo Fisher Scientific, Waltham, MA, USA) at 4000–500 cm^−1^, with a resolution of 2 cm^−1^, recording 32 scans.

#### 3.3.2. Elemental Analysis

The percentage content of nitrogen in the cellulose samples was analyzed with the Flash 2000 Series elemental analyzer (Thermo Fisher Scientific, Waltham, MA, USA). The results are expressed as the average values in triplicate measurements.

#### 3.3.3. X-ray Diffraction (XRD) Technique

The supermolecular structures of the cellulose samples were assessed through powder X-ray diffraction (XRD) analysis using a SmartLab X-ray diffractometer (Rigaku, Tokyo, Japan). Spectra were captured at 30 mA with an accelerating voltage of 40 kV using a Cu Kα radiation source. The diffraction pattern was recorded from 5° to 30° (2θ-angle range) with a step size of 0.04°/3 s. Peak deconvolution was carried out according to the method proposed by Hindeleh and Johnson [44] and subsequently re-fined and programmed by Rabiej [45]. After separating the XRD lines, the degrees of crystallinity (Xc) of the samples were determined by comparing the areas under the crystalline peaks and the amorphous curve.

#### 3.3.4. Dynamic Light Scattering (DLS) Method

The hydrodynamic diameter (particle size) of the nanocellulose samples was assessed using dynamic light scattering (DLS). Initially, the samples were dispersed in deionized water and sonicated for 25 min using a Sonic-2 ultrasound system (Polsonic Palczyński Sp. J., Warsaw, Poland, 230 V). The size characterization was performed at room temperature with a Zetasizer Nano ZS-90 (Malvern Instruments Ltd., Malvern, UK), and the results are reported as size distribution by intensity.

#### 3.3.5. Scanning Electron Microscopy (SEM) Technique

The morphology of the samples was analyzed using a Zeiss EVO 40 scanning electron microscope (Carl Zeiss AG, Oberkochen, Germany). Before the SEM analysis, the cellulose samples were coated with a thin layer of gold using a Balzers SCD 00 sputter coater (BalTec Maschinenbau AG, Pfäffikon, Switzerland).

#### 3.3.6. Statistical Analysis

Factorial one-way ANOVA followed by Tukey’s honest significant difference (HSD) test at α = 0.05 was performed using TIBCO Software Inc.’s Statistica version 13.3 (Palo Alto, CA, USA).

## 4. Conclusions

The results obtained in this study show that the antisolvent used to regenerate cellulose after the solvolysis reaction with 1-ethyl-3-methylimidazolium acetate affects the properties of cellulose. The results of the FTIR analysis and the analysis of nitrogen concentration in cellulose samples show that water, ethanol and a mixture of acetone and water successfully removed the used ionic liquid from the cellulose structure. The FTIR analysis showed that the spectrum of cellulose regenerated with acetone and acetonitrile contained peaks (C=N stretching vibrations, C-H bending vibrations and C=O stretching vibrations arising from IL), which confirmed the presence of ionic liquid in the regenerated cellulose samples. Moreover, the high IL content in the cellulose structure regenerated with these antisolvents was confirmed by the analysis of nitrogen content. The cellulose samples regenerated with acetonitrile and ethanol were characterized by a polymorphic structure of cellulose II, while the cellulose regenerated with water and a mixture of water and acetone presented a polymorphic structure of cellulose III. All cellulose samples showed a decrease in the crystallinity index compared to Avicel cellulose, and this decrease was related to the solvent used in cellulose regeneration. The decrease in the Xc index was also related to the remains of the ionic liquid in cellulose—the greater the amount of unremoved IL from the cellulose structure, the lower the Xc value. The water-regenerated cellulose had the lowest average particle size and was the only one that did not form agglomerates. Cellulose samples regenerated with other antisolvents were characterized by larger particle sizes and the formation of particle clusters. The formation of agglomerates by IL-treated cellulose was confirmed by SEM analysis.

Based on the results obtained, it can be concluded that, among the tested antisolvents, water leads to the effective removal of IL from the cellulose structure, which is additionally characterized by the smallest particle size and non-formation of agglomerates. Water’s greater effectiveness in cellulose regeneration may be related to its ability to form more hydrogen bonds with IL ions than the other antisolvents used. In addition, water has a smaller particle size than other solvents, so it can move between cellulosic polymers more easily. Acetate anions break hydrogen bonds in cellulose and are therefore more strongly attracted to water molecules with their stronger hydrogen bonds compared to other antisolvents. Moreover, the obtained results indicate that, by changing the conditions of cellulose regeneration (use of different antisolvents) after ionic liquid treatment, the properties of regenerated cellulose-based materials can be optimized.

## Figures and Tables

**Table 1:** The nitrogen content in cellulosic materials.

Symbol	N (%)
Avicel	nd
1 (acetone)	7.759 ^a^ ± 0.041
2 (acetonitrile)	4.520 ^b^ ± 0.245
3 (ethanol)	0.722 ^c^ ± 0.118
4 (water)	0.248 ^c^ ± 0.039
5 (acetone/water)	0.278 ^c^ ± 0.053

Values denoted with identical letters do not differ significantly at *p* = 0.005 according to the post hoc test, following one-way ANOVA test; nd—not detected.

**Table 2:** The Xc of cellulose samples.

Symbol	Xc (%)
Avicel	66
2 (acetonitrile)	29
3 (ethanol)	33
4 (water)	38
5 (acetone/water)	41

## Data Availability

Data are available from the corresponding author upon reasonable request.
